# Architecture of a consent management suite and integration into IHE-based regional health information networks

**DOI:** 10.1186/1472-6947-11-58

**Published:** 2011-10-04

**Authors:** Oliver Heinze, Markus Birkle, Lennart Köster, Björn Bergh

**Affiliations:** 1 Department of Information Technology and Medical Engineering, University Hospital Heidelberg, Speyerer Straße 4, 69115 Heidelberg, Germany

## Abstract

**Background:**

The University Hospital Heidelberg is implementing a Regional Health Information Network (RHIN) in the Rhine-Neckar-Region in order to establish a shared-care environment, which is based on established Health IT standards and in particular Integrating the Healthcare Enterprise (IHE). Similar to all other Electronic Health Record (EHR) and Personal Health Record (PHR) approaches the chosen Personal Electronic Health Record (PEHR) architecture relies on the patient's consent in order to share documents and medical data with other care delivery organizations, with the additional requirement that the German legislation explicitly demands a patients' opt-in and does not allow opt-out solutions. This creates two issues: firstly the current IHE consent profile does not address this approach properly and secondly none of the employed intra- and inter-institutional information systems, like almost all systems on the market, offers consent management solutions at all. Hence, the objective of our work is to develop and introduce an extensible architecture for creating, managing and querying patient consents in an IHE-based environment.

**Methods:**

Based on the features offered by the IHE profile Basic Patient Privacy Consent (BPPC) and literature, the functionalities and components to meet the requirements of a centralized opt-in consent management solution compliant with German legislation have been analyzed. Two services have been developed and integrated into the Heidelberg PEHR.

**Results:**

The standard-based Consent Management Suite consists of two services. The Consent Management Service is able to receive and store consent documents. It can receive queries concerning a dedicated patient consent, process it and return an answer. It represents a centralized policy enforcement point. The Consent Creator Service allows patients to create their consents electronically. Interfaces to a Master Patient Index (MPI) and a provider index allow to dynamically generate XACML-based policies which are stored in a CDA document to be transferred to the first service. Three workflows have to be considered to integrate the suite into the PEHR: recording the consent, publishing documents and viewing documents.

**Conclusions:**

Our approach solves the consent issue when using IHE profiles for regional health information networks. It is highly interoperable due to the use of international standards and can hence be used in any other region to leverage consent issues and substantially promote the use of IHE for regional health information networks in general.

## Background

IT-based inter-organizational and inter-sectoral communication in healthcare is required to accomplish the increasing demands of healthcare providers especially regarding quality of care and economical aspects. There exist different approaches and products to address this issue. Personal Health Record (PHR) and Electronic Health Record (EHR) systems are in widespread use in various projects around the world providing different advantages and disadvantages with respect to technical, ethical and data privacy issues as well as patient empowerment aspects. However, all approaches depend on the patient's consent in order to share documents and medical data with other care delivery organizations. The consent has to be given voluntarily on a well informed and competent basis. The patient has the right to know how his information is processed and who is able to access it and for which purposes. On one hand these requirements strengthen the autonomy of the patient implying a demand to participate in his healthcare. On the other hand the patient consent imposes new technical requirements on the PHR and EHR systems which are not yet implemented by most of the vendors. Even the ISO 20514 [1] does not address the consent issue although the problem has been discussed for several years as the existing literature reveals [2-8].

Similarly to other regions the Rhein-Neckar-Region with its 2.3 Million inhabitants aims to establish a regional health information network (RHIN). For this network the University Hospital Heidelberg designed a Personal Electronic Health Record (PEHR) architecture based on a service oriented architecture (SOA) according to profiles from the initiative Integrating the Healthcare Enterprise (IHE) and international standards like HL7 and DICOM [9,10]. The PEHR concept envisions a strong patient involvement to take patients' rights into account giving patients full control of the management of the access rights. The data privacy aspects and the management of access rights in this context have been discussed in depth earlier [11].

According to the German legislation, it is mandatory to provide consent mechanisms that comply with the opt-in principle in order to transfer medical data electronically between different institutions [12]. The regulations demand in particular that no data-transfer occurs prior to the patients' opt-in.

In order to address this issue for our regional network, theoretical considerations led to an abstract model for a consent management solution. Two implementable, practical approaches were derived from that model, a centralized and a decentralized approach [13].

With respect to a standardized implementation, the IHE Basic Patient Privacy Consent profile (BPPC) [14] provides opt-in support, but important aspects like e.g. how to structure a consent document and how to make the legal text machine-readable, are not specified. Hence, two additional services were needed in order to solve the consent issue in our RHIN, namely a Consent Creator Service (CCS) used by the patient to create a consent document and the so-called Consent Management Service (CMS) to manage the consent documents generated by the patients.

The present publication firstly describes a technical solution for a Consent Management Suite (COMS) with a centralized approach, related to the BPPC profile using IHE XDS.b, HL7 Version 3 Clinical Document Architecture (CDA) and the OASIS eXtensible Access Control Markup Language (XACML) and secondly an integration solution into IHE-based RHINs like the one in the Rhine-Neckar Region.

The objectives of this paper are:

• to describe and introduce an extensible and standard-based architecture for creating and managing patient consents (store and query electronically) in order to share medical data in an IHE XDS.b based PEHR scenario especially fulfilling the requirements of the opt-in approach

• to enrich the IHE-BPPC profile by formulating solutions for the undefined open aspects and to provide a practically usable solution which can potentially serve as a reference implementation

• to demonstrate how a centralized COMS can be integrated into an existing service oriented RHIN systems landscape.

### Integrating the Healthcare Enterprise (IHE)

Integrating the Healthcare Enterprise (IHE) is a joint initiative by healthcare professionals and manufacturers. The basic approach is that in a first step users design clinically feasible workflows supporting daily healthcare practice, which are in a second step transferred into a detailed technical specification. The results are grouped in so-called integration profiles consisting of actors and transactions. Actors can be implemented in systems like Hospital Information Systems (HIS) and communicate using transactions. For each transaction IHE defines which standard (e.g. HL7 or DICOM) should be used and how communication shall be implemented [15].

### Cross Enterprise Document Sharing (XDS.b) Profile

The Cross Enterprise Document Sharing (XDS) integration profile defines how to share medical documents across the boundaries of healthcare institutions like e.g. hospitals or physician's practices. The following actors are used in the context of our work: Document Source Actor and Document Consumer. These actors communicate via the following transactions with a Document Registry and Document Repository: Provide and Register Document Set-b transaction, Registry Stored Query and Retrieve Document Set transaction [16].

### Basic Patient Privacy Consents (BPPC) Profile

The BPPC profile [14] provides guidance on how to address consent aspects in IHE-based RHINs in a so-called XDS affinity domain (AD). An AD is an affiliation of e.g. healthcare providers in a region who have decided to share information of their jointly treated patients via e.g. shared-care records, i.e. a PEHR.

The present publication solely provides recommendations for XDS scenarios. BPPC provides the creation of a basic vocabulary of codes identifying the different privacy policies of which a consent document is made up. Such a policy should include the legal text (what should be shared, which users or roles may access the information and when, etc.) as well as a unique patient privacy consent identifier (OID). This OID should be used to mark published documents using the attribute "confidentialityCode". Opt-in scenarios can be implemented using a dedicated policy enforcing that nothing is to be shared prior to the patients' opt-in. When using XDS.b, like in our work, two actors are required for implementing BPPC: the Document Source Actor and its transaction Provide and Register Document Set-b (ITI-41) and the Document Consumer Actor with the transactions Retrieve Document Set (ITI-43) and Registry Stored Query (ITI-18). According to BPPC, these actors are to implement the Basic Privacy Enforcement Option as well as the Basic Patient Privacy Proof Option. The first option enforces that the XDS Registry has to validate whether all documents do contain a confidentialityCode from the AD vocabulary. The second enables a document consumer to query for Patient Privacy Consent Acknowledgement Documents with the document class 'consent'. The returned EventCodeList contains the information of underlying policies [17].

The following basic process is outlined in the profile:

• Recording a patient's acknowledgement of a privacy consent policy via Content Consumer Actor

• Checking for a patient's acknowledgement of a privacy consent policy (Basic Patient Privacy Proof Option)

• Publishing documents according to a consent policy (Document Source Actor has to check whether a policy exists allowing to publish the document. If yes, he has to set the OIDs to the confidentialityCode attribute of the document).

• Using published documents (The document consumer actor should enforce his own access control based on the returned EventCodeList)

In general, a consent document itself contains sensitive information. This should be taken into account within each affinity domain [14].

### HL7 Version 2 Medical Document Messages (MDM)

HL7 Medical Document Message (MDM) is a message type form the HL7 Version 2 family. MDMs are designed to transport medical documents either by reference or with content. The latter requires the encoding of the document in Base64 and adding the text string to the observation segment (OBX) [18].

### HL7 Version 2 Query/Response Conformance Statements

The HL7 Query/Response Conformance Statements provide a mechanism to build query messages in different ways. In our work, query by parameter (QBP) messages were used on the basis of the interrogative interaction model. This implies that the service receiving a query message has to respond with a response message [19].

### HL7 Version 3 Clinical Document Architecture (CDA)

The HL7 Clinical Document Architecture (CDA) is a document markup standard that specifies the structure and semantics of clinical documents in order to standardize them for exchange. A CDA document is defined as an information object which can include text, images and other multimedia content. Documents are encoded in XML. Meanings of single parts are derived from the HL7 Reference Information Model (RIM) and use the HL7 Version 3 data types. Major components are the header element, containing document meta data, and the body element containing the medical data. Three different levels are defined describing the degree of semantic interoperability reaching from plain text to fully structured and coded information. Two of the CDA's characteristics are human-readability as well as machine-readability [20].

### Extensible Access Control Markup Language (XACML)

The Extensible Access Control Markup Language is an XML-based OASIS standard. It provides syntax to define policies and specifies how to interpret these policies in order to allow or deny the requested action from a caller. Three major instances are defined which have to be implemented in a system environment using authentication on XACML-basis: The Policy Administration Point (PAP) is the system which manages the policies. The Policy Decision Point (PDP) is responsible to evaluate and issue authorization decisions. The Policy Enforcement Point (PEP) finally has to be implemented in the system which captures user's access request to a resource and enforces PDP's decision [21].

## Methods

Based on the capabilities and features offered by the IHE profile BPPC [14], previous work [13] as well as literature ([2-8,22-24]), the necessary functionalities and components to meet the requirements and workflows of a centralized opt-in consent management solution for IHE XDS.b based RHIN were designed and modeled using the Unified Modeling Language (UML).

In order to implement the two different services of COMS, Java was used as programming language and Apache Tomcat respectively the JBoss Application Server served as application server running both services.

For storing and querying the consent documents, the CMS was designed using HL7 MDMs to receive consent documents and HL7 QBP messages to query the CMS in order to receive particular consent information. The implementation was conducted with the open eHealth integration platform (IPF) from the Open eHealth Foundation [25]. For testing purposes, consents were stored in an open source IHE XDS.b Registry and Repository called openxds from the Open Health Tools project (OHT) [26] and the OHT Master Patient Index (MPI) was used to identify patients. Consents will later be stored in the Registry and Repository of the Heidelberg PEHR project.

In addition to BPPC and XDS.b profiles, XACML and HL7 CDA were used to build the consent documents with the CCS according to the patients' requests, which also renders them technically and semantically interoperable and ensures machine-readability of the consent documents. The identification of healthcare providers was achieved by a Provider and Organization Registry Service (PORS) developed in a prior Heidelberg project [27].

## Results

The first section is dedicated to a description of the insufficiently defined parts within the BBPC profile for opt-in scenarios and possible solution approaches, followed by sections on the COMS architecture, the interfaces, the integration into an IHE-based RHIN as well as its supported workflows according to IHE BPPC and the structure of the consent documents. Although the two services of COMS are designed to be used in networks of this kind, they can also be used separately in any other scenario that requires the management of consents e.g. the secondary use of clinical information.

### BPPC deficits for opt-in scenarios

As described above, BPPC provides many useful functions and recommendations to manage patients' consent documents. However, there is a gap between the provided functionality and the requirements for opt-in based consent management approaches which consists of three main issues:

• BPPC demands the usage of OIDs in the "confidentialityCode" field. At present there is no recommendation or specification on how to proceed with legacy documents lacking this attribute. Additionally, the Basic Patient Privacy Proof requires returning all privacy policies to the decentralized policy decision points, which could lead to privacy issues in certain circumstances.

• There is no workflow description for the management of consent documents.

• The so-called "advanced patient privacy consents", i.e. excluding a particular physician from accessing the information cannot be implemented with BPPC. A policy in the sense of BPPC identifies who has access to which information, but the mechanism for publishing this policy is not specified.

The first issue is addressed by not using confidentialityCodes for the patient's consent. Of course each transaction and the documents themselves are under a certain policy in an opt-in setting. Hence, a document consumer or a document source has to verify prior to the execution of a transaction whether it is allowed to conduct it or not. The second issue is solved by establishing a centralized policy decision point represented by the CMS, which is also responsible for the management of consent documents. The third issue is addressed by using XACML techniques embedded into the CDA policy document. Our approach is also applicable for opt-out scenarios.

The following sections describe the implemented COMS solution in detail as well as the integration into the Heidelberg PEHR.

### Architecture of COMS and integration into IHE-based environments

**Figure 1:**
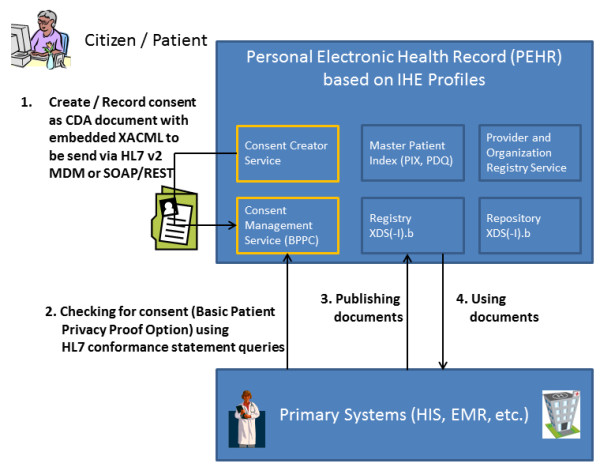
**Consent Management Suite (COMS) in the context of a regional health information network (RHIN)**. Overview of COMS and its services (yellow) in IHE-based regional health networks using the Heidelberg PEHR as an example.

The COMS consists of two different services (Figure 1). The CMS takes responsibility for receiving consent documents in a structured format (see paragraph consent document), for storing them and for handling consent queries from connected systems. In the Heidelberg PEHR project the CMS represents all three: the policy administration point (PAP), the policy decision point (PDP) and the policy enforcement point (PEP). The integration of those three elements into one service is due to the requirements of the German opt-in regulation. Otherwise, connected systems could unrightfully gain access to information which is not intended for them. The second service is the CCS. The patient uses this service to create consent documents according to the structured format described in the "consent document" paragraph. CCS can build the consent document dynamically by creating XACML-based policies using information from PORS containing all connected physicians and care delivery organizations. This approach allows advanced patient privacy consents. Figure 1 shows how both services, CMS and CCS, are integrated into the service oriented IHE-based architecture of the Heidelberg PEHR. The patient will use the CCS to manage his consent. The connected primary systems are using CMS to verify particular actions like e.g. publishing documents into the PEHR. The PEHR system itself will use CMS in order to acknowledge the access and review of documents for invoking physicians.

### Consent Management Service

**Figure 2:**
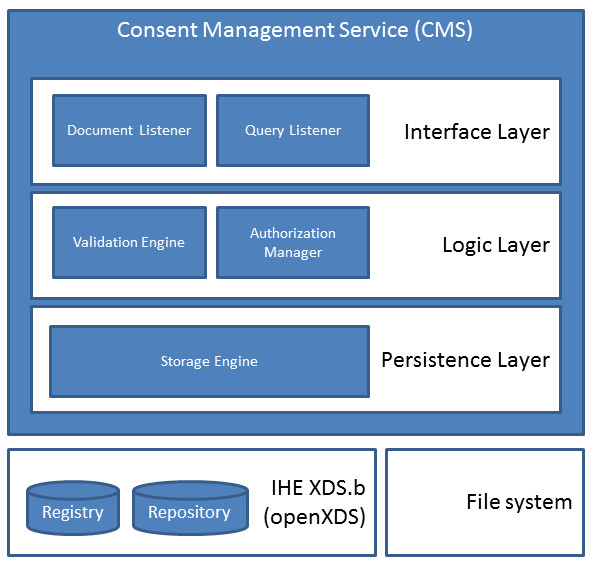
**Architecture of the Consent Management Service (CMS)**. The CMS has a three-layer architecture. The Interface Layer provides a document listener and a query listener. The first one receives consent documents and the latter queries for consent policies. The listener uses the validation engine from the Logic Layer in order to validate messages, documents and queries. The authorization manager is the core of CMS and implements the PDP and PEP. It uses the storage engine to fetch consent documents for a particular patient.

**Figure 3:**
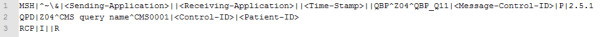
**Example of an HL7 Query to the Consent Management Service**. Queries are built using the HL7 Query/Response conformance statements. The message header (MSH) segment contains meta data including: the sending and receiving application, time stamps and versions. The essential part of the message is the query parameter definition (QPD) segment including the query characteristics. Possible values are defined and specified among the affinity domain members.

The CMS consists of a three-layer architecture (Figure 2). The interface layer provides a document listener and a query listener. The first one can receive consent documents via HL7 v2 MDMs or a SOAP web service both containing the CDA consent documents. The latter receives queries for consent policies using HL7 QBP messages. Figure 3 displays an example of such a query. Inside the query parameter definition (QPD) segment, actors have to enter their queries. Possible values are specified and implemented among the affinity domain members. The listener uses the validation engine from the logic layer in order to validate messages, documents and queries. The authorization manager is the core of CMS and implements the PDP and PEP. It uses the storage engine in order to get consent documents related to the appropriate patient from the query. According to the query, the authorization manager checks whether the requested action is allowed or not and processes the response. The storage engine is responsible for storing and retrieving consent documents either from an XDS.b Registry/Repository using the transactions ITI-41 and ITI-43 or from a file system depending on the configuration of the CMS.

### Consent Creator Service

The CCS is a Java-based tool providing a web-based interface in order to create a consent document based on CDA and XACML. The web-interface can be integrated into the context of a primary system like a HIS or like in our case into the patient view of the PEHR. A patient can create and edit the personal consent document in order to manage a) who has access to documents in the record, b) which primary systems, like hospital information systems or practice management systems can add new documents and c) which document types should be transferred to the PEHR. The patient can choose providers or organizations according to their hierarchy (organization → role→ person) from a dynamically generated list and allocate the desired rights. Document types are configured by administrators of the affinity domain. Patients can select on the basis of the document hierarchy in combination with a specific organization (document class → document type, e.g. referral letters → referral letters from cardiology) as well as exclude a dedicated document instance (e.g. referral letter from cardiology of University Hospital Heidelberg dated 15^th ^of August). The service creates an XACML-based policy-set according to the patient's choices and stores it as CDA document. The mapping of the patient's choices to an XACML representation is done on the basis of a deny based PEP and the combining algorithm "first-applicalble". For each hierarchy linree (Providers and Content) there is a policy inside the policy-set containing several rules. These rules are processed in a bottom-up approach from fine- to coarse-grained until a match occurs.

The completed consent document is sent to the CMS via an HL7v2 MDM for validation and storage. Consents can also be changed and updated in a similar way. The CCS provides interfaces to the MPI as well as to the PORS in order to identify patients, physicians and organizations correctly.

### The consent document

**Figure 4:**
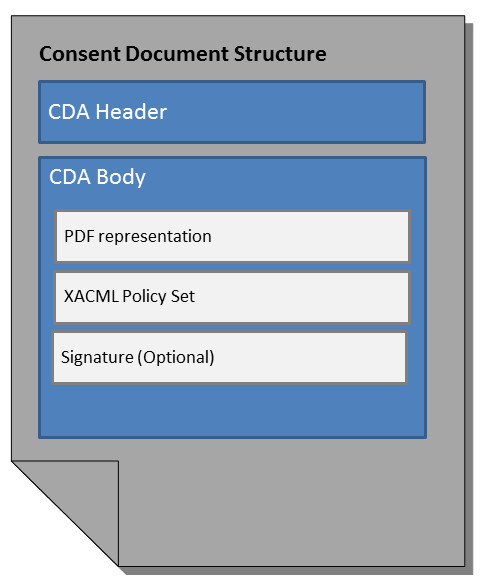
**Structure of the consent document**. The consent document is based on HL7 version 3 CDA. The header contains meta data about the patient (e.g. MPI-ID), the author and the legal authenticator. The body displays information like the involved providers and organizations as well as the consent information itself as assembled by the patient. The consent content (which person or organization is allowed to perform which transactions on which documents) is represented in an XACML policy set inside the body structure of the CDA document as well as in a PDF representation and can optionally have a digital or wet signature.

The consent document itself is an HL7 Version 3 CDA document based on the definitions of the BPPC profile (Figure 4). It contains the fundamental and universal text for consents related to a dedicated affinity domain and essential information about the patient (MPI-ID), the author, the legal authenticator, the involved providers and organizations (PORS-ID) as well as the consent rules chosen by the patient. These choices (which person or organization is allowed to perform which transactions on which documents) are technically characterized in policies, which are bundled in a policy set. These policies are represented in XACML inside the body structure of the CDA document. Each policy consists of a human-readable text describing the effect of the policy and a machine-readable section containing the coding of the policy for retrieving and processing. The tremendous advantage of CDA is to provide both, a human readable presentation of the document and a machine-readable version. The CDA document can also include a PDF transformation of the consent and optionally a digital signature. It can be printed for signature by the patient and for archiving purposes to ensure legal compliance.

### Integration of COMS into an XDS.b based setting

The integration of the COMS into an IHE XDS.b based setting is described in the following for the Heidelberg PEHR architecture which provides a PIX/PDQ-based MPI, an XDS.b Registry and Repository as well as a PORS. Three core workflows are required for handling consents in opt-in based IHE XDS.b scenarios: the creation of the consent, the inclusion of clinical documents into the PEHR and thirdly the access to those.

**Figure 5:**
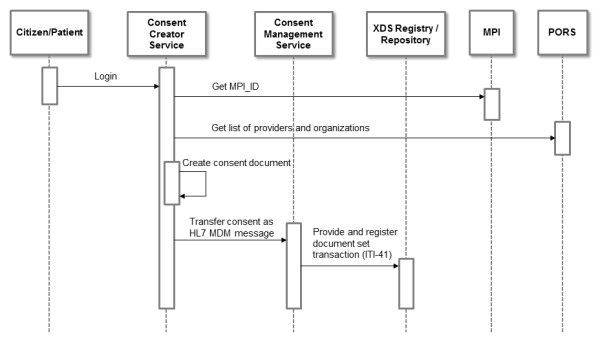
**UML sequence diagram of workflow 1: recording a patient's consent**. This UML sequence diagram shows the actors involved in recording a patient's consent with COMS in an integrated RHIN using the Heidelberg Personal Electronic Health Record architecture as an example.

The first workflow encompasses the recording of a patient's consent (Figure 5). The patient logs on to his PEHR which then interfaces to the GUI of the CCS allowing the patient to create or change his consent document. In a first step the CCS queries the MPI to receive the global identification of the patient (MPI-ID). Afterwards, it queries the PORS to obtain the latest and actual list of providers and organizations of the affinity domain. Then the patient can select which organization or which provider is allowed to review documents inside the PEHR and which system is granted to publish new documents to the record. The flexibility of XACML additionally offers the possibility to exclude specific document types. By the time the patient has finished his consent document, the CCS transfers the document to the CMS using an HL7 MDM message. The CMS extracts necessary meta data (e.g. MPI-ID) from the message and sends the document to the Registry and Repository using the IHE transaction ITI-41.

This is the trigger allowing documents to be published to the PEHR or clinical documents to be viewed using the PEHR according to the rules defined in the consent document.

**Figure 6:**
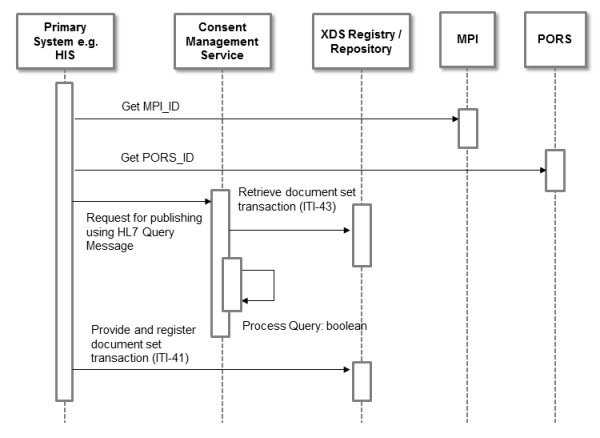
**UML sequence diagram of workflow 2: publishing documents by primary systems**. This UML sequence diagram demonstrates the actors involved in publishing documents with COMS in an integrated RHIN using the Heidelberg Personal Electronic Health Record architecture as an example.

Figure 6 demonstrates the second workflow: publishing documents by primary systems. Primary systems like e.g. HIS participating in the RHIN have to verify technically whether they are allowed to perform a particular transaction initiated by a healthcare professional. To obtain this consent information, primary systems connected to an affinity domain have to implement the Document Source Actor and the Content Creator Actor which queries the CMS. The primary system has to fetch the MPI-ID of the relevant patient and the PORS-ID of the inquiring organization and the inquiring physician in order to build an HL7 query conformance statement. This request is sent to the CMS which extracts the patient and loads the corresponding consent document from the repository using the transaction Retrieve Document Set (ITI-43). On the basis of the query and the content of the consent document, it generates the response message to the calling system. When publishing is allowed, the primary system can publish the document to the repository using the Provide and Register Document Set transaction (ITI-41). This consent verification process within the primary system is mandatory due to the German legislation which forbids the transfer of any kind of data prior to the patient's opt-in. Hence verifying the consent first within the PEHR would be illegal since patient data would leave the hospital environment without prior permission. However, some scenarios and settings may for security reasons require an additional verification process by the PEHR system itself.

**Figure 7:**
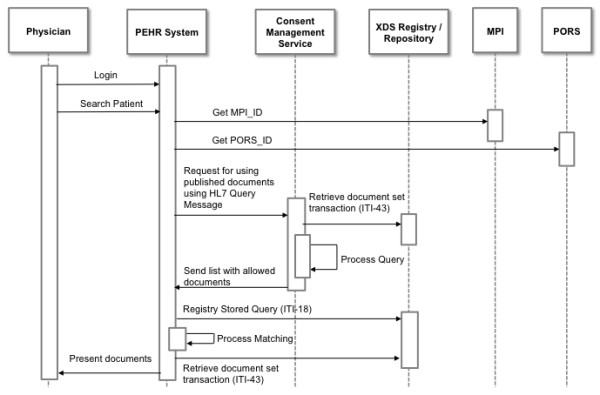
**UML sequence diagram of workflow 3: viewing published documents**. This UML sequence diagram shows the involved actors when viewing published documents with COMS in an integrated RHIN using the Heidelberg Personal Electronic Health Record architecture as an example.

The third and last workflow illustrates (Figure 7) how published documents can be reviewed using the PEHR. A physician logs on to the PEHR (either using the web frontend or via single sign-on from the primary system) and starts a search for a particular patient. The PEHR system knows the MPI-ID from its MPI and the corresponding PORS-ID from the logged-in user and implements the Document Consumer Actor as well as the Content Consumer Actor to generate an HL7 query message (QBP) in order to ask the CMS for permission. CMS retrieves the consent document from the repository using ITI-18 and ITI-43 and processes the consent against the query. In case allowance is generally granted, the CMS would return a list of allowed documents to the PEHR system via the HL7 QBP response. The PEHR would then request all allowed documents via ITI-18 from the Registry and retrieve the matching ones via ITI-43 from the Repository which are then presented to the physician in the PEHR GUI.

## Discussion

Our analysis identified the delta between the IHE BPPC profile definitions and the requirements of the German legislation for opt-in scenarios. With COMS we developed a method and a tool to overcome this gap by electronically recording a patient's consent using the IHE BPPC profile, CDA documents as well as XACML and demonstrated feasibility by integrating COMS into the PEHR of the Rhine-Neckar Region.

The selected technologies and tools have proven to be quite sufficient as they represent state-of-the-art technology and fit smoothly into the existing systems landscape and development environments. The usage of Open Source Software facilitated cost efficient and quick results. The employment of IHE profiles and worldwide standards like HL7 and XACML ensures technical interoperability not only within our own systems environment but also with other systems and hence the COMS approach and tools can easily be adapted by other regions according to their own settings and needs. However, from the authors' point of view the application of worldwide approaches like IHE is ambiguous; on one side they are without alternative for establishing technically interoperable systems in healthcare; on the other hand, due to their nature, they sometimes suffer from a lack of granularity on the conceptual level and with respect to definitions, demanding for customized solutions as outlined here.

Many existing solutions (e.g. [5,6]) have been explicitly designed and geared towards dedicated settings. In contrast to those, our standards-based approach of COMS represents a universally deployable architecture which benefits from its flexibility and can hence be used in any setting where the management of patients' consents is required. It is capable of managing advanced or non-advanced patient privacy consents for both opt-in and opt-out based RHIN. Also advanced consents beyond the scope of the BPPC profile can be generated by the patient dynamically via the CCS and can afterwards be validated by the CMS.

Other than proposed by the BPPC profile, the proposed solution can be used as centralized policy enforcement point, which is represented by CMS and which is essential for legal compliance in a German setting. Otherwise, connected systems could gain knowledge of consent policies they are not meant to know.

According to the German legislation, the transfer of any kind of data is forbidden unless the patient has opted-in. Thus, a proper opt-in has to be incorporated into the whole process as was described in the integration section. Another aspect is that we do not use confidentialityCodes in order to enforce the consent due to the limited nature of those codes. This offers the advantage of not touching all documents in a repository in the case of a revision of the patient consent. Furthermore, even old documents can be processed. The principle is that each patient has got several medical documents but always only one consent document. All medical documents underlie certain policies defined in the one consent document. The centralized PEP enforces these policies in contrast to several decentralized PEPs. Many primary systems (e.g. HIS) can process HL7 messages in order to communicate with the centralized PEP. The implementation, certification and maintenance of various decentralized PEPs is extremely sophisticated and costly in the long run, especially with RHIN increasing in size.

The proposed COMS is a stand-alone application which has pros and cons. One major set of advantages is that it can be used together with any off-the-shelf XDS.b implementation in otherwise proprietary settings as long as the interfacing to the COMS conforms with standards and supports various other use cases requiring patients' consents, like e.g. the clinical research domain. Its main disadvantage is the comparatively complex and extensive interfacing with a multitude of message interactions, which is obviously less fault tolerant and may present security issues. Alternatively the functionality of CMS can be directly integrated into an XDS.b Registry and Repository which would reduce the message complexity and can potentially improve the overall system performance.

Our present COMS implementation has some limitations. Security aspects like logging, authentication and authorization of the involved actors are not yet implemented due to the fact that the reference implementation of COMS is deployed in a closed network which secures all communications via secure socket layer inside a virtual private network. However, these elements will be addressed in future releases.

## Conclusions

The here proposed and developed architecture solves the consent issue for German RHINs by providing a legally compliant solution combinded with an efficient way of integrating primary systems into the network. Additionally it also supports opt-out scenarios, is very flexible and can be adapted to other settings in other regions worldwide. However, the COMS may not be considered an out of the box solution. Obviously an adaptation to the respective local requirements and rules of the affinity domain has to be undertaken but this can be achieved without complete redesign within the general framework of the proposed architecture by customization and parameterization. When privacy policies shall be generated dynamically, at least an MPI and a healthcare provider directory service like PORS are required.

As for today, many German projects including the ministerial ones mainly focus on proprietary architectures and communication protocols, often justified with deficits in existing global standards. We hope that our three main achievements, solving the consent issue for IHE, providing a blueprint for a standard-based architecture, and demonstrating its viability in the PEHR project in the Rhine-Neckar Region, all together will substantially promote the broader usage and deployment of IHE-based RHINs in Germany.

At present the COMS is in alpha release phase and will be open sourced with its first release candidate at the Open eHealth Foundation.

## Competing interests

The authors declare that they have no competing interests.

## Authors' contributions

OH conceived the project idea, was responsible for the architecture as well as the integration concept and wrote the manuscript. MB participated in the architecture, designed the HL7 messages and implemented the CMS. LK designed the consent document and implemented the CCS. BB reviewed the manuscript. All authors read and approved the final manuscript.

## Pre-publication history

The pre-publication history for this paper can be accessed here:

http://www.biomedcentral.com/1472-6947/11/58/prepub

## References

[B1] ISO ISO/TR 20514 Health informatics - Electronic health record - Definition, scope and context ISO/TR 20514:2005(E) 2005 ISO ed. Geneva Switzerland: ISO copyright office

[B2] Blobel B Authorisation and access control for electronic health record systems Int J Med Inform 2004 73 251 257 10.1016/j.ijmedinf.2003.11.018 15066555

[B3] Namli T Dogac A Implementation Experiences on IHE XUA and BPPC Technical Report Middle East Technical University Ankara 2006

[B4] Win KT Fulcher JA Consent mechanisms for electronic health record systems: a simple yet unresolved issue J Med Syst 2007 31 91 96 10.1007/s10916-006-9030-3 17489500

[B5] Heimly V Berntsen KE Consent-based access to core EHR information. Collaborative approaches in Norway Methods Inf Med 2009 48 144 148 19283311 10.3414/ME9214

[B6] Bergmann J Bott OJ Pretschner DP Haux R An e-consent-based shared EHR system architecture for integrated healthcare networks Int J Med Inform 2007 76 130 136 10.1016/j.ijmedinf.2006.07.013 16971171

[B7] Kluge EH Informed consent and the security of the electronic health record (EHR): some policy considerations Int J Med Inform 2004 73 229 234 10.1016/j.ijmedinf.2003.11.005 15066551

[B8] Neame R Olson MJ Security issues arising in establishing a regional health information infrastructure Int J Med Inform 2004 73 285 290 10.1016/j.ijmedinf.2003.11.010 15066560

[B9] Heinze O Bergh B Establishing a Personal Electronic Health Record in the Rhein-Neckar Region Informatica Medica Slovenica 2009 14 3 9 19745279

[B10] Heinze O Brandner A Bergh B Establishing a personal electronic health record in the Rhine-Neckar region Stud Health Technol Inform 2009 150 119 19745279

[B11] Bergh B Bach N Brandner A Heinze O Dössel O, Schlegel WC EHR access rights and the role of the patient IFMBE Proceedings World Congress on Medical Physics and Biomedical Engineering, September 7-12; Munich, Germany 2009 316 319

[B12] Meier A Der rechtliche Schutz patientenbezogener Gesundheitsdaten Münsterraner Reihe 84 2003 Karlsruhe: Verlag Versicherungswirtschaft21980538

[B13] Birkle M Heinze O Bergh B Schreier G, Hayn D, Ammenwerth E Entwurf eines elektronischen Einwilligungsmanagements für ein intersektorales Informationssystem eHealth 2010: Health Informatics meets eHealth 2010 OCG Books 264 Vienna: Österreichische Computer Gesellschaft

[B14] IHE IHE IT Infrastructure (ITI) Technical Framework Volume 1 Integration Profiles Revision 7 Basic Patient Privacy Consents Integration Profile 2010

[B15] IHE Welcome to Integrating the Healthcare Enterprise http://www.ihe.net last visited August 2011

[B16] IHE IHE IT Infrastructure (ITI) Technical Framework Volume 1 Integration Profiles Revision 7 Cross-Enterprise Document Sharing (XDSb) 2010

[B17] IHE IHE IT Infrastructure (ITI) Technical Framework Volume 2b Integration Profiles Revision 7 Basic Patient Privacy Enforcement Option 2010

[B18] HL7 HL7 Version 2.6 9 Medical Records/Information Management (Document Management) 2007

[B19] HL7 HL7 Version 2.5.1. Chapter 5 Query 2007

[B20] HL7 HL7 Clinical Documentation Architecture Release 2 2005

[B21] OASIS Extensible Access Control Markup Language 2005

[B22] Sujansky WV Faus SA Stone E Brennan PF A Method to Implement Fine-Grained Access Control for Personal Health Records Through Standard Relational Database Queries J Biomed Inform 2010 43 46 50 10.1016/j.jbi.2010.08.001 20696276

[B23] Sucurovic S Implementing security in a distributed web-based EHCR Int J Med Inform 2007 76 491 496 10.1016/j.ijmedinf.2006.09.017 17084662

[B24] Sucurovic S Milutinovic V The need for the use of XACML access control policy in a distributed EHR and some performance considerations Stud Health Technol Inform 2008 137 346 352 18560096

[B25] OeHF Open eHealth Foundation http://www.openehealth.org last visited August 2011

[B26] OHT Open Health Tools http://www.openhealthtools.org last visited August 2011

[B27] Heinze O Ihls A Bergh B Development of an Open Soruce Provider and Organization Registry Service for Regional Health Networks Third International Conference on Health Informatics (HealthInf 2010) 2010 Valencia, Spain 535 537

